# Towards Global HPV Eradication: Single-Dose HPV Vaccination vs. Pseudoscience

**DOI:** 10.3390/pathophysiology33020025

**Published:** 2026-03-30

**Authors:** Reona Shiro, Ikuo Tsunoda

**Affiliations:** 1 Department of Obstetrics and Gynecology, Kindai University Faculty of Medicine, Osaka 590-0197, Japan; reona00746@gmail.com; 2 Department of Microbiology, Kindai University Faculty of Medicine, Osaka 590-0197, Japan

**Keywords:** adverse reactions, anti-vaxxer, Cervarix, Gardasil, interleukin-17, macrophagic myofasciitis, poliomyelitis, uterine cervical neoplasm, vaccine hesitancy, World Health Organization

## Abstract

Human papillomavirus (HPV) can cause cervical cancer. Global viral eradication relies on specific criteria, including a single host species and effective vaccines, a feat successfully achieved with smallpox and rinderpest. Although measles is also a candidate for elimination, its progress has been hindered by vaccine hesitancy based on misinformation about vaccine safety. Similarly, HPV is an ideal candidate for eradication due to its strict human infectivity and the proven vaccine efficacy in reducing cancer rates and establishing herd immunity. We highlighted the growing global consensus on single-dose HPV vaccination to improve feasibility and compliance with comparable effectiveness and safety to three-dose vaccination. Supporting this, we demonstrated that mice receiving a single HPV vaccine produced anti-HPV antibodies without a prolonged pro-inflammatory cytokine profile. On the other hand, in Japan, a nine-year suspension of proactive government recommendations occurred due to alleged adverse events termed “HPV vaccination-associated neuro-immunopathic syndrome (HANS),” drastically reducing vaccination rates, despite rigorous international studies have confirmed the vaccine’s safety. Critical scientific evaluation demonstrated that HANS failed to meet the criteria for autoimmune diseases (Witebsky’s postulates); no evidence has been presented that HANS is a novel autoimmune disease. The claim of molecular mimicry between HPV L1 and human proteins was based solely on flawed computational analyses. Furthermore, the hypothesis implicating a pathogenic role for aluminum adjuvants was unsupported by experimental evidence; HANS animal models were flawed methodologically and unreproducible experimentally. In summary, we believe that implementing worldwide HPV vaccination strategies, including gender-neutral and single-dose programs, as well as denouncing pseudoscientific claims hold the potential to eliminate high-risk HPV types globally.

## 1. Introduction

### 1.1. Viral Eradication from the Earth by Vaccinations

For a worldwide microbial eradication, four criteria have been proposed: (a) a single host species (e.g., humans) without an animal or environmental reservoir; (b) accurate diagnostic tests; (c) effective and safe vaccines; and (d) long-period nationwide interruption of transmission. Although most pathogens do not meet criterion (a), several highly pathogenic viruses can infect only a single host, which can be prevented by vaccinations. To date, two viral diseases have been eliminated from the Earth through global vaccination programs: smallpox and rinderpest (Table 1).

Smallpox was caused by the variola virus (VARV), which is an enveloped, double-stranded DNA virus belonging to the genus *Orthopoxvirus*, family *Poxviridae*. Among the orthopoxviruses that infect humans, VARV was not zoonotic. In 1981, smallpox eradication was announced, and VRAV became the first virus eliminated from the Earth [1]. Rinderpest was the second viral disease eradicated worldwide through vaccination in 2001 [2]. It was characterized by fever, diarrhea, and high mortality and was caused by rinderpest virus (RPV). RPV was transmitted among cloven-hoofed animals, including cattle and buffalo [3]. RPV was an enveloped negative-sense single-stranded RNA virus belonging to the genus *Morbilivirus*, family *Paramyxoviridae*.

Among six viruses classified within the genus *Morbillivirus*, measles virus (MeV) can infect only humans, and its safest vaccines (MMR, measles, mumps, and rubella) have been available; thus, global elimination of MeV could be feasible. In 1998, however, Andrew Wakefield et al. published a manuscript suggesting that the MMR vaccine caused autism [4]. Although Wakefield’s manuscript was retracted due to serious flaws and Wakefield’s medical license was revoked, the original publication still caused public concerns about MMR vaccines, affecting the vaccination rate. In 2024, the annual number of measles deaths was 95,000 [5].

Poliomyelitis is another viral disease that can be eradicated and is caused by poliovirus (PV), a non-enveloped, positive-sense, single-stranded RNA virus belonging to the genus *Enterovirus*, family *Picornaviridae*. PV can infect only humans by an oral–fecal route, and effective oral poliovirus vaccine (OPV) and inactivated poliovirus vaccine (IPV) have been used worldwide. Although OPV could induce both humoral and cellular immune responses, including mucosal immunity, genetic reversion of the live attenuated poliovirus strains of OPV could result in the emergence of vaccine-derived poliovirus (VDPV), which is one of the reasons why poliomyelitis has not been eliminated from the Earth despite the global vaccination efforts [6].

Papillomavirus is a DNA virus belonging to the family *Papillomaviridae* and is highly species-specific: human papillomavirus (HPV) infects only humans with no other reservoir or vector [7]. Since current HPV vaccines are effective and safe, global HPV vaccinations can eliminate HPV types included in HPV vaccines. Indeed, HPV vaccinations have been reported to decrease the number of cervical cancers not only in vaccinated persons, but also in non-vaccinated persons, demonstrating the achievement of herd immunity against high-risk HPV types that cause cervical cancer.

### 1.2. HPV Genome, Proteins, and Types

HPV has a double-stranded, circular DNA genome encoding the structural late proteins L1 and L2 and the nonstructural early proteins E1, E2, and E4–E7. L1 and L2 are the major and minor capsid proteins, respectively; viral infection is initiated by L1 binding to host cells, after which HPV replicates within the nucleus [8]. E proteins play essential roles in viral replication, transcriptional regulation, immune evasion, and host cell-cycle manipulation, with E6 and E7 functioning as the primary oncogenic drivers in high-risk HPV types [9,10]. E6 binds to p53, a tumor suppressor protein, and targets it for rapid degradation via a cellular ubiquitin ligase [11]. E6 also induces cellular immortalization by activating telomerase and inhibits apoptosis by degrading pro-apoptotic proteins [12]. E7 interacts with the retinoblastoma protein (pRB) and promotes pRB degradation, leading to deregulated activity of the transcription factor E2F, and forces S-phase entry and genomic instability [12,13]. In high-risk HPV types, E6 and E7 act synergistically to enable persistent proliferation and malignant transformation. On the other hand, in low-risk HPV types, E6 does not bind p53, and E7 binds pRB with less affinity [7]. E6 and E7 in low-risk HPV types support productive viral replication without malignant transformation [14]. Moreover, E5, a small membrane-associated protein, contributes to early stages of carcinogenesis by enhancing growth factor signaling, modulating endosomal acidification, and suppressing antigen presentation, thereby promoting proliferation and immune evasion [15].

Papillomaviruses were first identified as small DNA viruses that could induce epithelial proliferative lesions in both animals and humans [16]. More than 200 HPV genotypes have been identified [17]. Among these genotypes, high-risk types such as HPV16, 18, 31, 33, 45, 52, and 58 were associated with cervical cancer [18]. HPV enters the host through micro-wounds in the skin or mucosal epithelium, caused by sexual activity or other mechanical stimuli, and infects basal cells in the deepest epithelial layers [19]. It is estimated that over 80% of sexually active women and men will acquire at least one HPV infection during their lifetime [20]. Although most HPV infections are cleared by the host immune system, persistent infection with high-risk HPV types in the cervix can lead to the development of cervical cancer [12].

### 1.3. Three HPV Vaccines, Adjuvants, and HPV Prevention

HPV vaccines are subunit vaccines that contain the L1 protein as an antigen [21]. The L1 protein can self-assemble into virus-like particles (VLPs) [22]. HPV vaccines also include adjuvants to enhance immunogenicity. Currently, three HPV vaccines are available: bivalent Cervarix^®^ (2vHPV), quadrivalent Gardasil^®^ (4vHPV), and nonavalent Gardasil^®^9/Silgard^®^9 (9vHPV). Adjuvants in 2vHPV are AS04 [composed of aluminum (Al) hydroxide (AH) and monophosphoryl lipid A (MPL)] [23], and those in 4vHPV and 9vHPV are Al hydroxyphosphate sulfate (AHS). 9vHPV covers nine HPV genotypes and can prevent almost 90% of cervical cancer cases globally [24].

All three HPV vaccines can prevent two high-risk HPV types, HPV16 and 18, which were the most frequently implicated in cervical cancer and have been associated with other genital cancers, including vaginal, vulvar, and penile cancers, as well as non-genital cancers, including oropharyngeal and anal cancers [25]. Although oropharyngeal cancer, which is the second most common HPV-associated cancer after cervical cancer, occurs more frequently in men, the number of cases in the United States has now surpassed that of cervical cancer [26]. Thus, infections with high-risk HPV types represent a significant carcinogenic risk factor not only for women but also for men. Infection with two low-risk HPV types, HPV6 and 11, can be prevented by 4vHPV and 9vHPV vaccinations; HPV6 and 11 infections can cause anogenital warts, benign lesions known as condyloma acuminata [27]. Presence of vulvar or vaginal condyloma acuminata in the mother during delivery may result in the development of juvenile-onset recurrent respiratory papillomatosis in the child [28].

HPV vaccines have been incorporated into national immunization programs in many countries and have demonstrated effectiveness in reducing HPV infection, precancerous lesions, and cervical cancer incidence [29,30,31]. Several countries with high vaccination coverage have been shown to be close to achieving elimination of cervical cancer [32,33]. Currently, the World Health Organization (WHO) encourages a gender-neutral program and supports a single-dose schedule to ensure equitable and expanded distribution of HPV vaccines [34].

### 1.4. Global Strategy to Eliminate Cervical Cancer

In recognition of the global burden of cervical cancer, the WHO launched “the global strategy to accelerate the elimination of cervical cancer as a public health problem” in 2020 [35]. The strategy is anchored in three key pillars: primary prevention through HPV vaccination; secondary prevention through screening; and treatment of precancerous lesions and invasive cancer through timely diagnosis. Cervical cancer screening has been recommended for the early detection of cervical cancer. Specifically, the WHO has set the 90–70–90 targets to be achieved by 2030: 90% of girls fully vaccinated with the HPV vaccine by age 15 years; 70% of women screened using a high-performance test by ages 35 years and again by 45 years of age; and 90% of women identified with cervical disease receiving appropriate treatment and care. Achieving these targets could prevent millions of cervical cancer cases and deaths worldwide over the coming decades. The three pillars of elimination hold different levels of importance. Primary prevention through vaccination forms the foundation of cervical cancer control. When vaccination rates are acceptable within a system, the sustainability of the cervical cancer prevention framework is assured. However, eliminating cervical cancer is impossible with inadequate HPV vaccination coverage. Thus, the success of the elimination strategy depends heavily on high HPV vaccination rates, as vaccination provides long-lasting population-level protection. In countries with low screening participation, strengthening HPV vaccination programs is expected to be especially effective at accelerating progress toward elimination.

Progress toward the WHO cervical cancer elimination targets has varied among countries and regions. In high-income countries (HICs), substantial variation in HPV vaccinations exists. Several HICs with early implementation of school-based HPV vaccination programs, such as Australia, the United Kingdom, and Nordic countries, have already achieved high vaccine coverage and substantial reductions in HPV prevalence, cervical intraepithelial neoplasia, and cervical cancer incidence. Australia, in particular, was projected to reach cervical cancer elimination thresholds within the next decade [33]. Differences in vaccination policy (female-only versus gender-neutral programs), public confidence in vaccine safety, screening uptake, and the health system have led to divergent trajectories toward elimination. Since HPV-related diseases, including cervical cancer, are sexually transmitted diseases, gender-neutral HPV vaccination (vaccinating both males and females) was more effective than female-only strategies at achieving rapid herd immunity and eradicating high-risk HPV types [36]. By reducing transmission in both sexes, these programs will provide stronger, more equitable protection, preventing HPV-related cancers also in men, such as oropharyngeal and anal cancers, and reducing the overall prevalence of the virus in the population.

Many low- and middle-income countries (LMICs), which account for the majority of global cervical cancer cases and deaths, will continue to face major barriers to implementation, including limited access to HPV vaccines, insufficient screening infrastructure, and constraints in diagnostic and treatment capacity. As a result, disparities in cervical cancer burden between HICs and LMICs were expected to widen in the absence of accelerated global investment and equitable vaccine distribution. Meanwhile, several LMICs, where the number of clinicians collecting pap smear samples is limited, have implemented HPV-based screening strategies using vaginal and urine self-sampling. The self-collected samples have been shown to achieve sensitivity comparable to that of clinician-collected samples, with improved participation in screening [37,38].

### 1.5. Single-Dose HPV Vaccination: Efficacy and Safety

With accumulating evidence on the efficacy of a single-dose HPV vaccination [39,40], the WHO Strategic Advisory Group of Experts on Immunization (SAGE), the principal advisory group to the WHO, has endorsed a single-dose HPV vaccination as an acceptable alternative for immunocompetent adolescents, particularly in resource-constrained settings [34]. This policy shift aimed to improve vaccine equity, expand coverage, and accelerate progress toward the global goal of cervical cancer elimination. Several countries, including the United Kingdom and Australia, have begun implementing or piloting single-dose schedules within national immunization programs [41,42]. Most recently, Kreimer et al. [43] reported that one dose of either 2vHPV or 9vHPV provided protection against HPV16 or HPV18 infection and was noninferior to two doses. Broader coverage achieved through simplified schedules may outweigh any marginal reductions in individual-level immunity.

In theory, a single-dose vaccination has another advantage over a three-dose vaccination, since it would induce a milder inflammatory reaction, resulting in fewer adverse events. We examined the hypothesis using a murine experimental system.

## 2. Materials and Methods

### 2.1. Mouse Experiments

We used female C57BL/6 mice from CLEA Japan, Inc. (Tokyo, Japan) and injected 5-week-old mice intramuscularly with 50 μL of 2vHPV, 4vHPV, or phosphate-buffered saline (PBS) as a control in the right quadriceps muscle. The mice received HPV vaccines or PBS three times on days 0, 28, and 56 (three doses) or only once on day 0 (single dose). We collected sera on days −7, 1, 14, 29, 42, and 57 by submandibular approach and on day 84 from the heart. All mice were maintained in the specific pathogen-free animal facility at Kindai University Faculty of Medicine, Osaka, Japan. All experimental procedures were approved by the Institutional Animal Care and Use Committee of Kindai University Faculty of Medicine (KAME-2022-001) and performed according to the criteria outlined by the National Institutes of Health (NIH) National Research Council.

### 2.2. Anti-HPV 16 L1 IgG Antibody Enzyme-Linked Immunosorbent Assay (ELISA)

Anti-HPV 16 L1 antibodies were quantified by enzyme-linked immunosorbent assay (ELISA) using sera. Flat-bottom 96-well plates (Thermo Fisher Scientific Inc., Waltham, MA, USA) were coated with 1 μg/mL of recombinant HPV16 L1 protein (Abcam plc., Cambridge, UK), incubated with serially diluted standards HPV 16 L1 monoclonal IgG antibody (Thermo Fisher Scientific Inc.) and serum samples, followed by horseradish peroxidase (HRP)-conjugated anti-mouse IgG (H + L) (Thermo Fisher Scientific Inc.). The immunoreactive complexes were detected with the BD OptEIATM TMB Substrate Reagent Set (BD Biosciences, Milpitas, CA, USA), and absorbance was measured at 450 nm using the Synergy H1 Hybrid Multi-Mode Microplate Reader (Agilent Technologies, Inc., Santa Clara, CA, USA) [44].

### 2.3. Cytokine Bead Arrays

We quantified the serum cytokines with the LEGENDplex™ Mouse Inflammation Panel(BioLegend, San Diego, CA, USA) [13-plex; interferon (IFN)-β, IFN-γ, CCL2/monocyte chemotactic protein (MCP)-1, tumor necrosis factor (TNF)-α, interleukin (IL)-1β, IL-6, IL-10, IL-12p70, IL-17A, IL-23, IL-27, and granulocyte-macrophage colony-stimulating factor (GM-CSF)] (Biolegend, CA, USA). Cytokine concentrations were measured by flow cytometry using the BD FACS Canto II (BD Biosciences, Milpitas, CA, USA) and analyzed with LEGENDplex™ Data Analysis Software Suite [44].

## 3. Results

### Anti-HPV Antibody and Cytokine Productions in Single-Dose Vaccinated Mice

**Figure 1:**
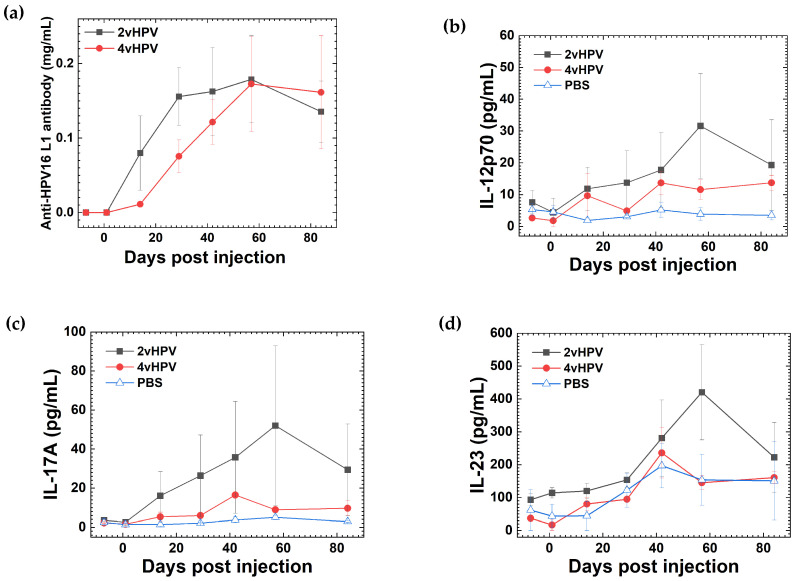
Serum anti- HPV16 L1 antibody and cytokine concentrations in experimental mice after a single injection of bivalent HPV vaccine (2vHPV, Cervarix^®^) or quadrivalent HPV vaccine (4vHPV, Gardasil^®^) on day 0. We quantified antibody and cytokine concentrations on days −7, 1, 14, 29, 42, 58, and 84 post-injection (p.i.) by enzyme-linked immunosorbent assay (ELISA) and cytokine bead arrays, respectively. (**a**) Anti-HPV antibodies were detected 2 weeks p.i. and peaked at 2 months p.i. (**b**) IL-12p70, (**c**) IL-17A, and (**d**) IL-23 concentrations increased continuously, reached the highest levels at 1.5 or 2 months p.i., and then declined by 3 months p.i. Values are the mean ± the standard error of the mean (SEM) from three to four mice per group. Control mice received phosphate-buffered saline (PBS) and had no detectable serum anti-HPV antibody.

We monitored serum cytokine concentrations in experimental mice receiving 2vHPV or 4vHPV every 4 weeks for three times [44]. Among 13 cytokines quantified by cytokine bead arrays, we found that concentrations of several cytokines, including IL-12p70, IL-17A, and IL-23, increased continuously over the observation period, reaching the highest levels at the end of the observation (Supplementary Figure S1) [44]. To determine whether the increasing levels of these cytokines could be attributed to the long-term effect of a single-dose HPV vaccination or to the additive effect of a three-dose HPV vaccination, we administered a single dose of 2vHPV or 4vHPV and monitored serum cytokine levels over the long observation period. We found that, in both 2vHPV and 4vHPV single-dose vaccinated mice, anti-HPV 16 L1 antibody titers were detectable 2 weeks after injection and reached the highest levels in 2 months (Figure 1a). On the other hand, concentrations of pro-inflammatory cytokines, including IL-12p70, IL-17A, and IL-23, peaked at 1.5 or 2 months post-injection and then declined by the end of the observation period (Figure 1b–d and Figure S1). These results showed that increased cytokine levels were transient after a single-dose HPV vaccination. Thus, in the mouse model, a single-dose HPV vaccination did not prolong cytokine elevation, implying a potentially safer profile for human HPV vaccination as well.

## 4. Discussion

The above findings, along with accumulated human epidemiological data, showed that single-dose vaccination is an effective and safe strategy for the global eradication of high-risk HPV types. Here, scientifically, it appears that global HPV elimination could be achievable in the near future. However, even in HICs with the capacity to meet the WHO elimination targets, progress has been slowed by anti-vaccine misinformation rooted in pseudoscience. In this section, we will discuss the situation in Japan, where the anti-vaccine movement has been prominent.

### 4.1. HPV Vaccine Crisis in Japan

**Figure 2:**
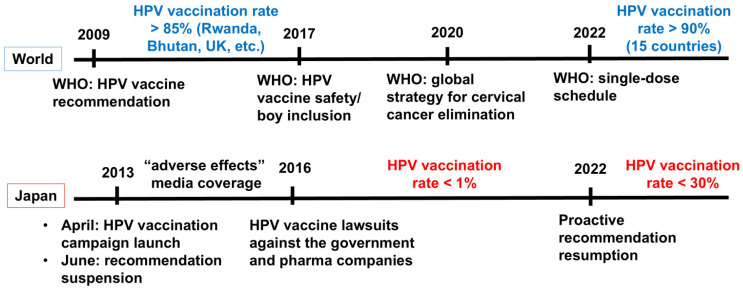
Key events and milestones of the HPV vaccination in the world and Japan. Abbreviations: HPV, human papillomavirus; WHO, World Health Organization.

Japan reports approximately 10,000 new cervical cancer cases and 3000 deaths annually [45]. Cervical cancers can be prevented by cancer screening and HPV vaccinations. Although screening participation rates in other HICs are approximately 80%, Japan’s rate was only about 40% in 2022, considerably lower than in other countries [46,47]. On the other hand, initially, the HPV vaccination campaign in Japan, launched in April 2013 with 2vHPV and 4vHPV for females aged 12–16, was promising, achieving a vaccination rate exceeding 70%. However, media coverage of alleged “diverse symptoms” as adverse events after vaccination led to a nine-year suspension of proactive government recommendations [48]. The HPV vaccination rate declined sharply to less than 0.1%, exposing an entire generation of young women to otherwise preventable cancer risk. With accumulating safety data on HPV vaccines, proactive recommendations for HPV vaccination have been resumed in Japan since 2022. However, the HPV vaccination rate in Japan has remained low due to a lack of public confidence in the safety of the HPV vaccine [49], and the incidence and mortality of cervical cancer in Japan have been increasing [50]. Epidemiological projections estimate thousands of additional cervical cancer cases and deaths attributable to the decline in vaccination rate [51,52] (Figure 2). Sekine et al. reported that the Japanese female age group, whose HPV vaccination rate declined during the suspension of proactive recommendations, had a significantly higher HPV16/18 infection rate compared with earlier age groups with high HPV vaccination coverage [41].

The Global Advisory Committee on Vaccine Safety (GACVS), established by the WHO, has repeatedly concluded that no causal association exists between HPV vaccination and the alleged “diverse symptoms.” During the vaccination suspension in Japan, information on neuropsychological adverse events following HPV vaccination was provided by a small group of neurologists who proposed a new disease entity, “HPV vaccination-associated neuroimmunopathic syndrome (HANS),” claiming that HPV vaccination caused diverse neurological and immunological symptoms. The original “proposed preliminary diagnostic criteria” for HANS was published only as a conference abstract [53] and has never been accepted internationally or published as a full-length scientific manuscript in peer-reviewed journals. Most reports of “victims” with HANS were published as essays or commentaries, not as original articles, in non-peer-reviewed commercial journals in Japanese or predatory journals. Only a few manuscripts have been accepted by peer-reviewed journals, and in all of them, the authors stated that the causal relationship between HPV vaccination and clinical signs/symptoms was not confirmed [54].

### 4.2. No Evidence for HPV Vaccinations as the Cause of Autoimmune Disease

Autoimmune etiology of human diseases can be established when the diseases meet the four criteria, named Witebsky’s postulates, which were modelled on Koch’s postulates as follows [55,56]. First, demonstration of autoantibodies or self-reactive T cells in the blood. Second, the identification of specific autoantigens that these autoantibodies or self-reactive T cells recognize. Third, circumstantial clinical evidence suggesting autoimmunity, including (a) medical history or family history associated with autoimmune diseases, (b) lymphocyte infiltration in the targeted organ, (c) association with specific major histocompatibility complex (MHC), and (d) disease amelioration by immunosuppression. Fourth, reproduction of the autoimmune disease in experimental animals: (a) by transfer of human pathogenic antibodies or pathogenic T cells to experimental animals; (b) by autoantigen injections into experimental animals; (c) by transfer of antibodies or lymphocytes from autoimmune animals into naïve animals; or (d) reproduction of the autoimmune diseases in genetically modified animals (Table 2). Based on the four criteria above, HANS cannot be regarded as an autoimmune disease. Among any reports including commercial publications by HANS-advocating neurologists, (1) neither autoantibodies/self-reactive T cells nor (2) their corresponding autoantigens specific for HANS were commonly identified in HANS cases. (3) In most HANS cases, there were no clinical data suggesting autoimmune diseases: for example, MHC association with HANS cases was denied; immunosuppressive therapy or plasmapheresis was ineffective in most cases or conducted without setting control cases. (4) No animal experiments reproduced HANS-like disease by any approaches, including HPV vaccinations, in experimental animals (see below, Section 4.4). Thus, there was no evidence supporting HANS as an autoimmune disease. Although some autoimmune diseases did not meet all four criteria of Witebsky’s postulates, none failed to meet any criteria, unlike HANS.

### 4.3. Critical Evaluation of HANS Hypotheses/Findings: Molecular Mimicry Hypothesis

As discussed above, no clinical evidence has supported that HPV vaccinations induce autoimmunity. However, in the ongoing HPV vaccination adverse events lawsuit in Japan, HANS proponents submitted previously published hypotheses/findings as evidence to explain plausible pathomechanisms of HANS [57]. There were three hypotheses/findings: (1) molecular mimicry between HPV L1 and human proteins; (2) pathogenicity of Al-containing adjuvants; and (3) animal models of HANS.

Molecular mimicry is a phenomenon in which antimicrobial antibodies or T cells trigger autoimmune responses against host cells or organs by cross-reacting with host molecules, particularly proteins, based on morphological similarities between microbial and host molecules. HANS proponents argued that previous molecular mimicry studies discovered HPV amino acid (AA) sequences in human proteins, which could cause organ damage by autoantibodies produced after HPV vaccinations [57]. As we previously criticized [58], all molecular mimicry analyses of HPV vaccines have been published only by Darja Kanduc’s group. They used computational analyses to show that a large number of human proteins shared 5–8 length AA sequences with full-length HPV proteins [59] and HPV16 L1 epitopes [60,61]. They have then hypothesized that HPV vaccinations could induce autoantibodies cross-reacting with human proteins, which would cause systemic lupus erythematosus, multiple sclerosis (MS), amyotrophic lateral sclerosis, diabetes, and sudden death [61,62,63].

Theoretically, if the full-length AA sequence of the microbial epitope matches a human protein, the protein could harbor a computationally validated epitope and the potential to elicit cross-reactive autoantibody production following infection or vaccination. On the other hand, if only in silico analysis showed that the portion of the epitope shared AA sequences with host proteins, the “partial” molecular mimicry alone would likely not result in the production of cross-reactive antibodies. Recently, we examined the antibody epitopes of HPV16 L1 protein, as in the studies by Kanduc’s group [60], and found that none of the entire sequences of any linear epitopes of HPV16 L1 were identical to any human protein AA sequences [64]. Thus, this study again demonstrated that computational analysis did not provide evidence that antibodies to the HPV16 L1 antigen could cross-react with human proteins.

On the other hand, organ damage by molecular mimicry has been proposed in several immune-mediated diseases and microbial infections, including rheumatic fever and Group A *Streptococcus* [65], Guillain–Barré syndrome and *Campylobacter jejuni* [66], MS and Epstein–Barr virus (EBV) [67,68], and type 1 diabetes and coxsackievirus [69]. In all cases, to propose the potential pathogenicity of molecular mimicry, cross-reactivities of antibodies or T cells should be proved experimentally: for example, anti-microbial antibody binding to human molecules using ELISA and Western blotting, or to human cells or tissue sections by immunohistochemistry; anti-microbial T cell killing of host cells or lymphoproliferation against human molecules. The presence of such cross-reactive antibodies and T cells should be further confirmed for their pathogenicity by the adoptive transfer of immune effector cells into naïve animals, to determine whether the transfer causes autoimmune organ damage. For examples, Fujinami and Oldstone [70] first reported that molecular mimicry between myelin basic protein (MBP) and viral proteins could induce cross-reactive antibody and T cell responses and that sensitization of the viral peptides with molecular mimicry resulted in immune cell infiltration in the brain of experimental rabbits, whose pathology was similar to the animal model for MS, experimental autoimmune encephalomyelitis (EAE). Later, anti-MBP T cells have been shown to be pathogenic by adoptive transfer experiments; cross-reactive antibodies were not pathogenic, since MBP is not expressed on the surface of the myelin sheath. Thus, the presence of the short AA sequences between microbes and host proteins alone should not be regarded as pathogenic molecular mimicry.

More recently, using ELISA and Western blotting, Lanz et al. [68] reported that monoclonal antibody against EBV derived from an MS patient had a cross-reactivity against glial cell adhesion molecule (GlialCAM), which is expressed in the central nervous system (CNS) by astrocytes and oligodendrocytes. Although the anti-EBV antibody recognizes the intracellular (but not extracellular) domain of GlialCAM, the authors suggested that molecular mimicry between EBV and GlialCAM may play a role in MS. The central epitope (EBV EBNA1_AA394-399_) of the anti-EBV antibody was composed of six AAs (PPRRPP); the first three AAs and the fifth AA were identical to those of GlialCAM (PPRAPS). Here, this molecular mimicry would not have been discovered if one had conducted a computational analysis looking for it at the 5AA- or 6AA-length level. This study was another example showing that experimental evidence of molecular mimicry was necessary to find molecular mimicry between microbes and host proteins.

### 4.4. Adjuvant Hypothesis and HANS Animal Model

HANS proponents argued that Al-adjuvants could induce immune dysregulation, leading to systemic and neurological immune-mediated diseases (“adjuvant hypothesis”) [57] (Table 3). Matsumura et al. [71] evaluated the adjuvant hypothesis and demonstrated that Al adjuvants have a decades-long safety record and that different adjuvants included in 2vHPV and 4vHPV should not induce the same syndrome, HANS. If Al-adjuvants were the cause of HANS, similar signs/symptoms should have appeared following vaccination with other vaccines containing the same adjuvants as 2vHPV and 4vHPV, which had not been observed.

HANS-pathogenesis was also explained by the induction of “macrophagic myofasciitis (MMF),” which has been reported only in France and proposed by a single research group. MMF was a novel disease entity that Al-containing vaccines could result in local accumulation of Al-laden macrophages in the vaccinated muscle, leading to immune-mediated diseases in remote organs, including the brain [72,73,74]. The pathogenic role of macrophage accumulation proposed in MMF cases has been refuted by animal studies, which demonstrated that Al-containing vaccine injections induced Al-laden macrophage accumulation only at the injection sites, without inducing systemic or brain pathology or neurological deficits [75,76]. Most recently, we demonstrated that 2vHPV and 4vHPV injections in mice induced only local macrophage accumulation at the injection sites, without neurological or systemic organ damage. Similar macrophage accumulation was observed in mice receiving other anti-viral vaccines containing Al-adjuvants. These results demonstrated that MMF was not a new disease entity, but a physiological reaction to Al-adjuvants [44].

**Table 3:** Pathogenic HPV vaccine hypotheses/findings and their counterevidence.

Hypotheses/ Findings	Claims	Counterevidence	Refs
molecular mimicry hypothesis	Sequence homology between HPV and human proteins should induce autoantibody	No human protein had identical full-length AA-sequences of any HPV epitopes	[58,64]
adjuvant hypothesis	Al-adjuvant could induce a novel disease, macrophagic myofasciites	Macrophage accumulation was not pathogenic, but a physiological reaction	[44,71,75]
animal model	HPV vaccine injection could induce neurological abnormalities in mice	HPV vaccine injection induced no pathology in mice	[44,48,58]

Abbreviations: AA, amino acid; Al, aluminum; HPV, human papillomavirus.

Retracted manuscripts and unpublished data from HANS-advocating groups reported that experimental HPV vaccination in mice caused behavioral and motor abnormalities, antibody deposition, microglial activation in the hippocampus (Inbar R et al. 2016, retracted by *Vaccine*), and atresia of the third ventricle (Aratani S et al., 2016, retracted by *Sci Rep*) (Table 3). However, none of these findings were reproducible by others [44].

## 5. Conclusions

Virologically, since HPV infects only humans and has no reservoir, high-risk HPV types can be eliminated globally. From a public health perspective, HPV elimination could be accelerated effectively and safely by implementing worldwide HPV vaccination strategies, including gender-neutral and single-dose programs. Lastly, denouncing pseudoscientific claims will help address the public concerns about alleged “adverse effects” following HPV vaccinations, which is also essential for global HPV elimination.

## Figures and Tables

**Table 1:** Viral diseases and their global elimination.

Viral Disease	Pathogen	Host	Vaccine		Eradication Status	Issue
smallpox	variola virus (VARV)	human only	○	vaccinia virus	eradicated (1981)	two frozen stocks
rinderpest	rinderpest virus (RPV)	cattle buffalo	○	TCRV	eradicated (2001)	14 RVCM
measles	measles virus (MeV)	human only	○	MMR	11 million cases 95,000 deaths (2024)	anti-vaccine hoax
poliomyelitis	poliovirus (PV)	human only	○	OPV IPV	41 cases (in Afghanistan and Pakistan) (2024)	circulating VDPV
cervical cancer	human papillomavirus (HPV)	human only	○	2vHPV 4vHPV 9vHPV	660,000 cases 350,00 deaths (2022)	anti-vaccine hoax

Abbreviations: ○; vaccine is available; 2vHPV/4vHPV/9vHPV, bivalent/quadrivalent/nonavalent HPV vaccine; IPV, inactivated poliovirus vaccine; MMR, measles, mumps, rubella vaccine; OPV, oral poliovirus vaccine; RVCM, rinderpest virus-containing material; TCRV, tissue culture rinderpest vaccine; VDPV, vaccine-derived poliovirus.

**Table 2:** Four criteria for autoimmune diseases: Witebsky’s postulates.

Criteria	Examples	Autoimmune Disease	HANS
1. autoreactive Ab or T cell	anti-neuromuscular junction Ab in MG	present	absent
2. specific autoantigen	acetylcholine receptor in MG	identified	no
3. clinical autoimmune data	(a) history, (b) tissue cell infiltration, (c) MHC link, (d) immune therapy	yes	no
4. reproduction in animals	(a) human Ab/T-cell transfer, (b) active induction, (c) passive Ab/T-cell transfer, (d) genetically modified animals	yes (e.g., EAMG)	no

Abbreviations: Ab, antibody; EAMG, experimental autoimmune myasthenia gravis; HANS, HPV vaccination-associated neuro-immunopathic syndrome; MG, myasthenia gravis; MHC, major histocompatibility complex.

## Data Availability

The original contributions presented in this study are included in the article/Supplementary Materials. Further inquiries can be directed to the corresponding author.

## Supplementary Materials

The following supporting information can be downloaded at: https://www.mdpi.com/article/10.3390/pathophysiology33020025/s1, Figure S1: Serum cytokine concentrations in mice receiving bivalent HPV vaccine (2vHPV, Cervarix^®^) or quadrivalent HPV vaccine (4vHPV, Gardasil^®^).

## Abbreviations

The following abbreviations are used in this manuscript:

2vHPV	bivalent HPV vaccine
4vHPV	quadrivalent HPV vaccine
9vHPV	nonavalent HPV vaccine
AA	amino acid
Ab	antibody
Al	aluminum
AH	aluminum hydroxide
AHS	aluminum hydroxyphosphate sulfate
CNS	central nervous system
EAMG	experimental autoimmune myasthenia gravis
EBV	Epstein–Barr virus
EAE	experimental autoimmune encephalomyelitis
ELISA	enzyme-linked immunosorbent assay
GACVS	Global Advisory Committee on Vaccine Safety
GlialCAM	glial cell adhesion molecule
GM-CSF	granulocyte-macrophage colony-stimulating factor
HIC	High income country
IL	interleukin
IFN	interferon
IPV	inactivated poliovirus vaccine
HANS	HPV vaccination-associated neuro-immunopathic syndrome
HPV	human papillomavirus
LMICs	low- and middle-income countries
MBP	myelin basic protein
MCP	monocyte chemotactic protein
MeV	measles virus
MG	myasthenia gravis
MHC	major histocompatibility complex
MMF	macrophagic myofasciitis
MMR	measles, mumps, and rubella
MPL	monophosphoryl lipid A
MS	multiple sclerosis
NIH	National Institutes of Health
OPV	oral poliovirus vaccine
PBS	phosphate-buffered saline
p.i.	post-injection
PV	poliovirus
RPV	rinderpest virus
SAGE	Strategic Advisory Group of Experts on Immunization
SEM	standard error of the mean
TNF	tumor necrosis factor
VDPV	vaccine-derived poliovirus
VLP	virus-like particle
VARV	variola virus
WHO	World Health Organization
