# Supplementary material for: Towards Global HPV Eradication: Single-Dose HPV Vaccination vs. Pseudoscience
Source: Pathophysiology. 2026 Mar 30;33(2):25. doi: 10.3390/pathophysiology33020025 (PMC13108217; doi:10.3390/pathophysiology33020025)
Supplement: Supplementary file 1 [file pathophysiology-33-00025-s001.zip › pathophysiology-4157204-supplementary.pdf]

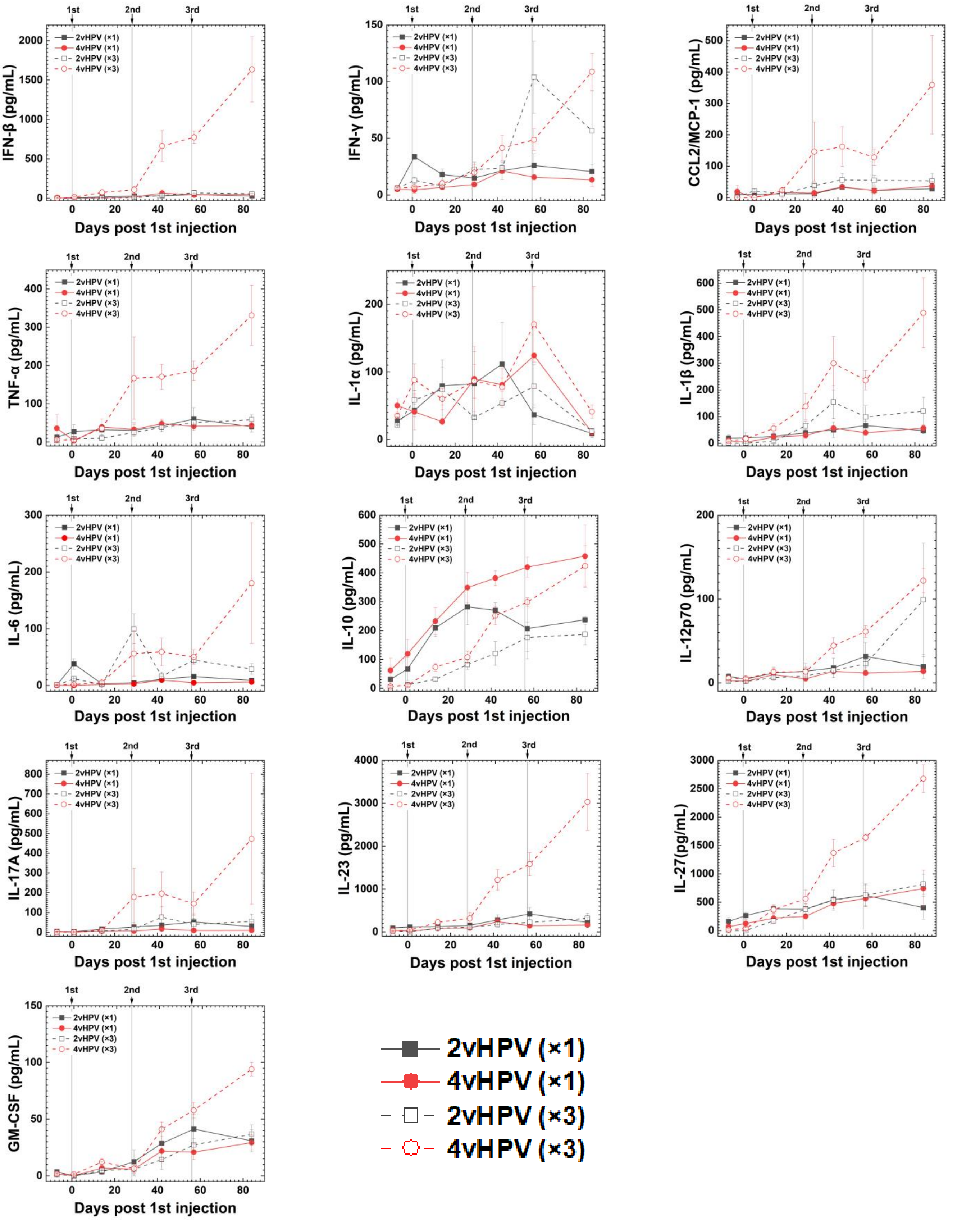

Supplemental Figure S1. Serum cytokine concentrations in mice receiving bivalent HPV vaccine (2vHPV, Cervarix®) or quadrivalent HPV vaccine (4vHPV, Gardasil®). We injected 2vHPV or 4vHPV intramuscularly into C57BL/6 mice every four weeks for three times (x3) or once on day 0 (x1). We quantified the serum cytokines using the LEGENDplex Mouse Inflammation Panel [13- plex; interferon (IFN)- β, IFN-γ, CCL2/monocyte chemotactic protein (MCP)-1, tumor necrosis factor (TNF)-α, interleukin (IL)-1β, IL-6, IL-10, IL-12p70, IL-17A, IL-23, IL-27, and granulocyte-macrophage colony-stimulating factor (GM-CSF)] (Biolegend, CA, USA) on days −5, 1, 14, 29, 42, 58, and 84 post-injection (p.i.). In mice receiving three 4vHPV, several cytokine concentrations, including IL-12p70, IL-17A, and IL-23, increased continuously during the observation period, reaching the highest levels at the end of the observation. On the other hand, in mice receiving a single injection of 2vHPV or 4vHPV, IL-12p70, IL-17A, and IL-23 concentrations reached the highest levels 1.5 or 2 months p.i. and then decreased 3 months p.i . Values are the mean ± the standard error of the mean (SEM) from three to four mice per group.
